# A New Dressing for Equino-Varus

**Published:** 1869-07

**Authors:** N. Senn

**Affiliations:** Ashford, Wis.


ARTICLE XXVI.

### A NEW DRESSING FOR EQUINO-VARUS.

By N. SENN, M.D., Ashford, Wis.

Having lately treated a case of equino-varus with a new dressing, the like of which I never saw, heard or read of, and having obtained with it a good result, it perhaps merits a few words of description on account of its simplicity.

The patient was a youth, 14 years of age; the deformity having existed for seven years, gradually increasing, until at the time when treatment was commenced he was hardly able to walk across the room without some kind of support. The muscles of the leg, especially the anterior, were extremely atrophied; the heel raised nearly two inches, the foot resting on the outer side of the dorsum and the external malleolus, the toes and foot turned inwards from contraction of the flexor brevis digitorum and fassia plantoris, the astragalus partially dislocated, forming quite a prominence on the anterior aspect of the ankle. While the patient was under the influence of an anæsthetic, I divided at once the tendo achillis, flexor brevis digitorum and fassia plantaris, in the usual manner, then flexed the foot forcibly, so as to bring it back as nearly as possible into the normal position, and placed it upon a posterior splint for the first day. At that time the small openings of the subcutaneous wounds were closed, and as there was hardly any pain or tenderness, I applied at once the following dressing: I had a shoe made just high enough to embrace the heel firmly, lined in front from the toes to the ankle; between the two soles was placed a piece of sheet iron cut in the shape and size of the sole of the foot, projecting in front in a narrow strip, to which could be applied a short strap with a buckle; on the outside of the heel was applied another short strap provided also with a buckle; next, two strips of adhesive plaster, three inches wide above and one below, were applied from just below and in front of the knee, where they met, and of sufficient length to reach to the upper part of the thigh, supported by circular strips and a bandage; to the lower end of the adhesive strips, two leather straps were attached, punctured by a sufficient number of holes to reach to the buckles in front and on the side. By lacing the shoe firmly, the curving of the foot could be gradually overcome, and a firm hold obtained; with the anterior strap the toes could be raised and the heel depressed; with the outside strap the foot could be turned outwards, thus fulfilling every indication. The straps can be shortened to any extent, so as to bring the foot in any position desired. With this dressing the patient was allowed to walk about on the second day, and never experienced any inconvenience whatever. He has now been under treatment four weeks. The foot is now almost in the normal position, and the muscles of the leg are gaining rapidly in size and strength. This dressing can be made use wherever there is a shoemaker and blacksmith, and with it almost every case of equino-varus can be treated successfully, provided the necessary anatomical obstacles are first removed with the knife.

